# Effect of delayed palliative chemotherapy on survival of patients with recurrent ovarian cancer

**DOI:** 10.1371/journal.pone.0236244

**Published:** 2020-07-23

**Authors:** Seung Yeon Pyeon, Gwan Hee Han, Kyung Do Ki, Kwang-Beom Lee, Jong-Min Lee

**Affiliations:** 1 Department of Obstetrics and Gynecology, Kyung Hee University Hospital at Gangdong, Seoul, Republic of Korea; 2 Department of Obstetrics and Gynecology, Gachon University Gil Medical Center, Gachon University College of Medicine, Inchon, Republic of Korea; Roswell Park Cancer Institute, UNITED STATES

## Abstract

For patients with recurrent ovarian cancer, the goals of chemotherapy include palliation of disease-related symptoms with minimum treatment-related side effects. However, there is currently a paucity of data regarding the initiation of palliative chemotherapy. This study aimed to compare the differences in survival rates and toxicities between patients with recurrent ovarian cancer who started palliative chemotherapy immediately versus those who received delayed chemotherapy. Through a retrospective chart review, patients who received more than three lines of chemotherapy were included. Based on the timing of third-line chemotherapy initiation, the patients were divided into two groups: delayed (DTG) and immediate (ITG) treatment groups. The chi-square test or Fisher’s exact tests, and t-test or Mann-Whitney U test were used for comparing variables, as appropriate. The Kaplan-Meier method was used for survival analysis. P-value of <0.05 was considered significant. Although there was no statistically significant difference, the total number of regimens and cycles was lower in the DTG than in the ITG. No differences in toxicities and survival rates were observed between the two groups. Overall, survival and toxicity did not differ significantly between the two groups. In a palliative care setting, our findings suggest that delaying the treatment had no adverse effect on survival. Despite the lack of evidence of a survival benefit with aggressive treatment, patients chose to continue chemotherapy. Because recurrent ovarian cancer is a complex condition, patients require sufficient explanation and time to fully understand the costs and benefits related to aggressive chemotherapy.

## Introduction

Ovarian cancer is an aggressive malignancy and the seventh most common cancer globally, with a 5-year survival rate of <45% [1]. Chemotherapy following cytoreductive surgery is the primary treatment for ovarian cancer, regardless of cancer stage; generally, ovarian cancer has a good response to chemotherapy. Despite optimal initial treatment, the majority of patients eventually develop recurrence. According to the World Health Organization’s GLOBOCAN 2012 project, 75%–80% of patients who successfully responded to initial treatment showed recurrence [1].

Patients with recurrent ovarian cancer receive palliative chemotherapy. In those with especially rapid recurrent cancer growth, severe cancer-associated symptoms are often experienced. With effective palliative chemotherapy, a decrease is observed in the severity of these symptoms. However, chemotherapy-related toxicity cannot be ignored. Various side effects are observed during chemotherapy depending on the regimen used and the response to the medications. These side effects not only affect a patient’s quality of life, but also their compliance with treatment [2]. For recurrent or refractory ovarian cancers, the goals of chemotherapy include palliation of disease-related symptoms and improvement of quality of life with minimum chemotherapy-related side effects [3]. However, limited data are available regarding the optimal timing for initiation of palliative chemotherapy [4].

Most trials on relapsing ovarian cancers are limited to patients receiving second- or third-line treatment, and there are no standardized treatment guidelines [5]. For patients in whom palliative chemotherapy is unsuccessful, the disease should be considered chronic. In other words, alleviation of symptoms becomes the primary goal rather than increasing overall survival; thus, treatment might be intentionally delayed to reduce the associated toxicity. This study aimed to compare the differences in survival rates and toxicities between patients with recurrent ovarian cancer who started palliative chemotherapy immediately versus those who received delayed palliative chemotherapy.

## Materials and methods

### Study design

We performed a retrospective chart review of patients with recurrent ovarian cancer who underwent primary surgical treatment at two centers (Kyung Hee University Hospital, Gangdong and Gachon University Gil Medical Center) from 2006 to 2016.

### Study participants and data collection

During this period, 214 patients were diagnosed with ovarian cancer at both hospitals; this number also included patients who were diagnosed with primary peritoneal carcinoma and fallopian carcinoma. All patients underwent cytoreductive surgery, and all surgeons attempted to minimize the size of the residual lesions. Subsequently, postoperative adjuvant therapy was administered according to the relevant guidelines. Assessment of disease recurrence was based on radiological findings. When abdominal computed tomography (CT) showed a new lesion after the completion of chemotherapy or disease progression during chemotherapy, the next line of chemotherapy was considered. Each physician determined the initiation timing of the next line of chemotherapy, based on the clinical symptoms, will of the patients to be treated, and effective drugs available. At both centers, the physicians who decided to start chemotherapy immediately started it within 2 weeks at the latest. In contrast, when the physicians decided to delay chemotherapy, the delay was as long as possible.

Patients who received at least three lines of chemotherapy were included in the current analysis. The timing of third-line chemotherapy initiation was used to divide the patients into two groups. The first group, the immediate treatment group (ITG), consisted of patients who started chemotherapy <3 weeks after the detection of recurrence or progression, as defined by the radiological findings. The second group, the delayed treatment group (DTG), included patients who started chemotherapy ≥3 weeks after the detection of recurrence or progression. Patients who responded to platinum-based chemotherapy and subsequently showed relapse ≥6 months after treatment were classified as being “platinum-sensitive.” Patients who showed relapse within 6 months of completing platinum-based chemotherapy were classified as being “platinum-resistant.” Platinum-resistance was assessed in all instances in which platinum-based chemotherapy was administered.

We recorded patient demographic characteristics such as age, body mass index (BMI), initial findings at diagnosis (such as cancer stage, histology, and residual volume after cytoreductive operation), clinical remission, and progression-free survival (PFS). The recurrence site was reviewed upon detection of a second recurrence or progression. Additionally, we reviewed the total number of chemotherapy regimens and cycles following the initiation of third-line chemotherapy. In administering each chemotherapy regimen, we calculated the duration from the diagnosis of recurrence or progression, based on imaging findings, to the initiation of chemotherapy. These durations (third-, fourth-, or fifth-line etc.) were summed together for the analysis. The toxicities associated with treatment were also assessed. If the patient had toxicity at least once during several lines of chemotherapy treatments, the toxicity according to grade was recorded. Anemia, thrombocytopenia, neutropenia, and febrile neutropenia were considered as hemodynamic toxicities. The occurrence of alopecia, sensory neuropathy, pulmonary toxicity, and allergic reactions was also recorded.

### Statistical analysis

Categorical variables were analyzed using the Chi-square test or Fisher’s exact test, and continuous variables were analyzed using the t-test or Mann-Whitney U test, as appropriate. The Kaplan-Meier method and log-rank test were used for survival analysis. A p-value <0.05 was considered significant. All analyses were performed using SPSS software version 24.0 (SPSS Inc., Chicago, IL).

### Ethics statement

This retrospective study was approved by the local institutional review boards (IRB) of the two hospitals (the Kyung Hee University Hospital at Gangdong IRB and the Gachon University Gil Medical Center IRB). To protect the patients’ privacy, all data were completely anonymized before analysis. The IRBs approved our study with waiver of informed consent because this study involved no risk to the patients and no interventions. This study did not have a predetermined protocol.

## Results

**Fig 1:**
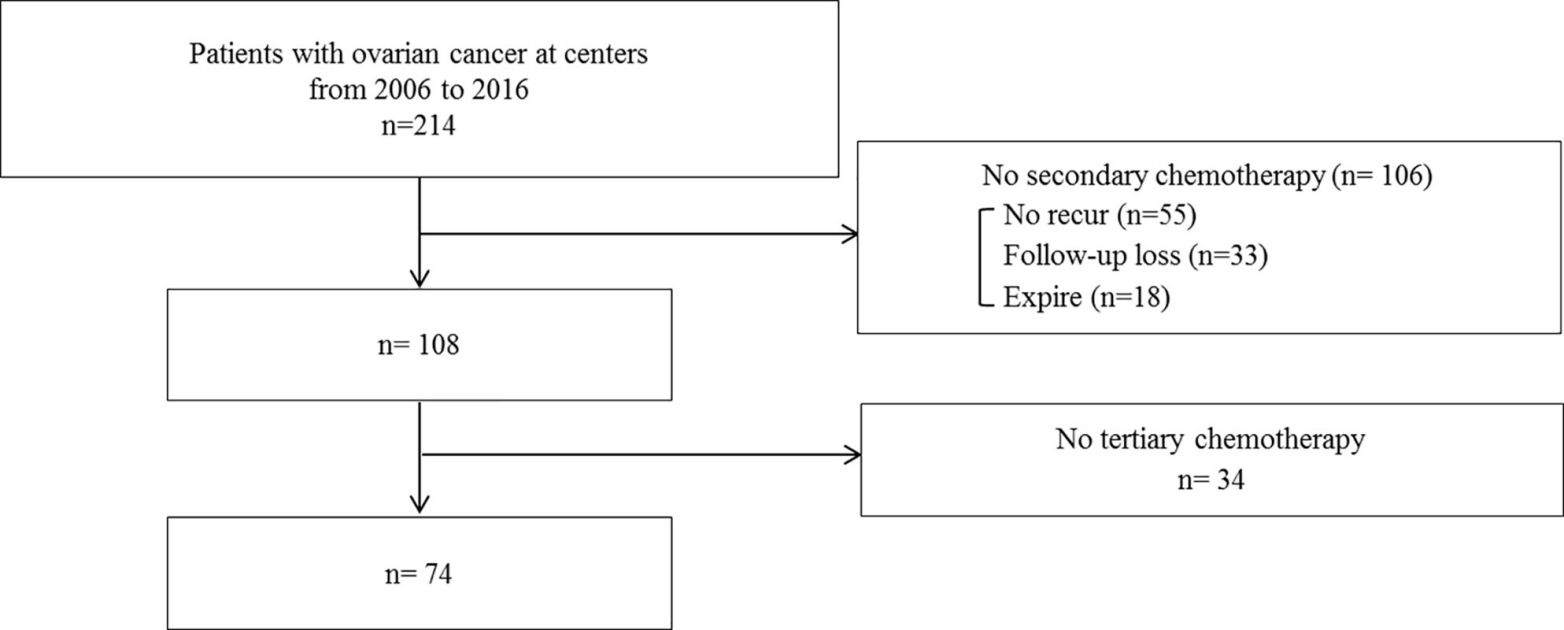
Flow diagram of patient selection.

A total of 214 patients were diagnosed with ovarian carcinoma at the two centers. Among them, a total of 74 patients received three or more lines of chemotherapy (Fig 1). The ITG included 44 women and the DTG included 30. The characteristics of patients are summarized in Table 1. The median age in the ITG and DTG was 53 years (range, 20–77 years) and 56 years (range 28–74 years), respectively. The median BMI in both groups was 23 kg/m^2^. The performance status at the time of diagnosis in all included cases was Eastern Cooperative Oncology Group status 0 or 1. The ITG included the following cases: 26 serous adenocarcinomas; 4 mucinous adenocarcinomas; 5 endometrioid adenocarcinomas; 5 clear cell adenocarcinomas; 1 mixed cell type; 2 undifferentiated types; and 1 yolk sac tumor. The DTG included the following cases: 18 serous adenocarcinomas; 4 mucinous adenocarcinomas; 2 endometrioid adenocarcinomas; 2 clear cell adenocarcinomas; 2 undifferentiated types; 1 squamous cell carcinoma and 1 transitional cell carcinoma. The initial characteristics at the diagnosis of recurrence did not differ significantly between the two groups.

**Table 1:** Characteristics of the patients.

		ITG (n = 44)	DTG (n = 30)	P value
Age, median(range)		53 (20–77)	56 (28–74)	0.569
BMI, median(range)		23 (16–32)	23 (17–32)	0.840
Stage, N (%)	I	7 (16.7)	3 (10.0)	
	II	5 (11.9)	2 (6.7)	
	III	18 (42.9)	20 (66.7)	
	IV	12 (28.6)	5 (16.7)	0.279
Histology, N (%)	S+E*	30 (70.5)	20 (66.7)	
	Others**	13 (29.5)	10 (33.3)	0.730
Residual volume at 1^st^ debulking operation, N(%)	<1	30 (78.9)	17 (60.7)	
	≥1	8 (21.1)	11 (39.3)	0.106

ITG, immediate treatment group; DTG, delayed treatment group; N, number; BMI, body mass index.

* S, serous adenocarcinoma; E, endometrioid adenocarcinoma.

** Mucinous adenocarcinoma, clear adenocarcinoma, transitional cell carcinoma, mixed cell type, undifferentiated type, yolk sac tumor, squamous cell carcinoma.

The median number of chemotherapy regimens in the ITG and DTG was 4 and 3, respectively, and the median number of chemotherapy cycles since third-line chemotherapy in the ITG and DTG were 8 and 6, respectively. For adjuvant chemotherapy (1^st^ line chemotherapy), 68/74 patients (92%) were treated with Paclitaxel + carboplatin (TC) regimen. 6 cases of Non-TC regimen included topotecan + cisplatin, belotecan single, two cases of carboplatin single, belotecan + etoposide + cisplatin in ITD group, and carboplatin single in DTG group. After recurrence, there are diversities of regimens. The median value of the sum of the duration from diagnosis to chemotherapy initiation was 3 (range, 0–46) weeks and 12 (range, 3–81) weeks, respectively. Resistance to platinum-based chemotherapy did not differ significantly between the two groups (ITG, 75% and DTG, 90%; p = 0.106) (Table 2).

**Table 2:** Characteristics of chemotherapy.

		ITG (n = 44)	DTG (n = 30)	P value
Recurrence site at 3^rd^ line of CTx, N (%)	Pelvic+peritoneum	17 (45.9)	16 (53.3)	
	Ascites+distant	20 (54.1)	14 (46.7)	0.548
Recurrence site at 3^rd^ line CTx, N (%)	Single site	18 (48.6)	14 (48.3)	
	Multiple site	19 (51.4)	15 (51.7)	0.976
N of total regimens	Median (range)	4 (3–9)	3 (3–8)	0.027
N of CTx cycles since 3^rd^ line of CTx				
	Median (range)	8(1–39)	6 (1–66)	0.076
Sum of duration from progression to start of CTx, wks	Median (range)	3 (0–46)	12 (3–81)	**0.000**
Platinum resistance at 3^rd^ line of CTx, N (%)	Sensitive	11 (25)	3 (10)	
	Resistance	33 (75)	27 (90)	0.106

ITG, immediate treatment group; DTG, delayed treatment group; N, number; CTx, chemotherapy.

The worst grade of toxicity per patient across multiple treatment regimens was compared. Table 3 shows the number of patients in each group who developed each grade of toxicity. No difference in toxicity was observed between the two groups.

**Table 3:** Toxicities.

			ITG (n = 44)	DTG (n = 30)	P value
Anemia	G1-2	N (%)	24 (54.5)	20 (66.7)	0.526
	G3-4	N (%)	12 (27.3)	7 (23.3)	
Thrombocytopenia	G1-2	N (%)	24 (54.5)	17 (56.7)	0.721
	G3-4	N (%)	7 (10.9)	3 (10.0)	
Neutropenia	G1-2	N (%)	20 (45.5)	18 (60.0)	0.214
	G3-4	N (%)	19 (43.2)	9 (30.3)	
Febrile neutropenia		N (%)	7 (15.9)	5 (16.7)	1.000
Alopecia	G1-2	N (%)	18 (40.9)	15 (50.0)	0.379
	G3-4	N (%)	24 (54.5)	13 (43.3)	
Sensory neuropathy	G1-2	N (%)	36 (81.8)	24 (80.0)	0.918
	G3-4	N (%)	8 (18.2)	5 (16.7)	
Ototoxicity		N (%)	2 (4.5)	1 (3.3)	1.000
Nephrotoxicity		N	4 (9.1)	2 (6.7)	1.000
Pulmonary toxicity		N	3 (6.8)	1 (3.3)	0.642
Allergic reaction		N	4 (9.1)	2 (6.6)	1.000

ITG, immediate treatment group; DTG, delayed treatment group; N, number of patients who experienced toxicity at least once.

**Fig 2:**
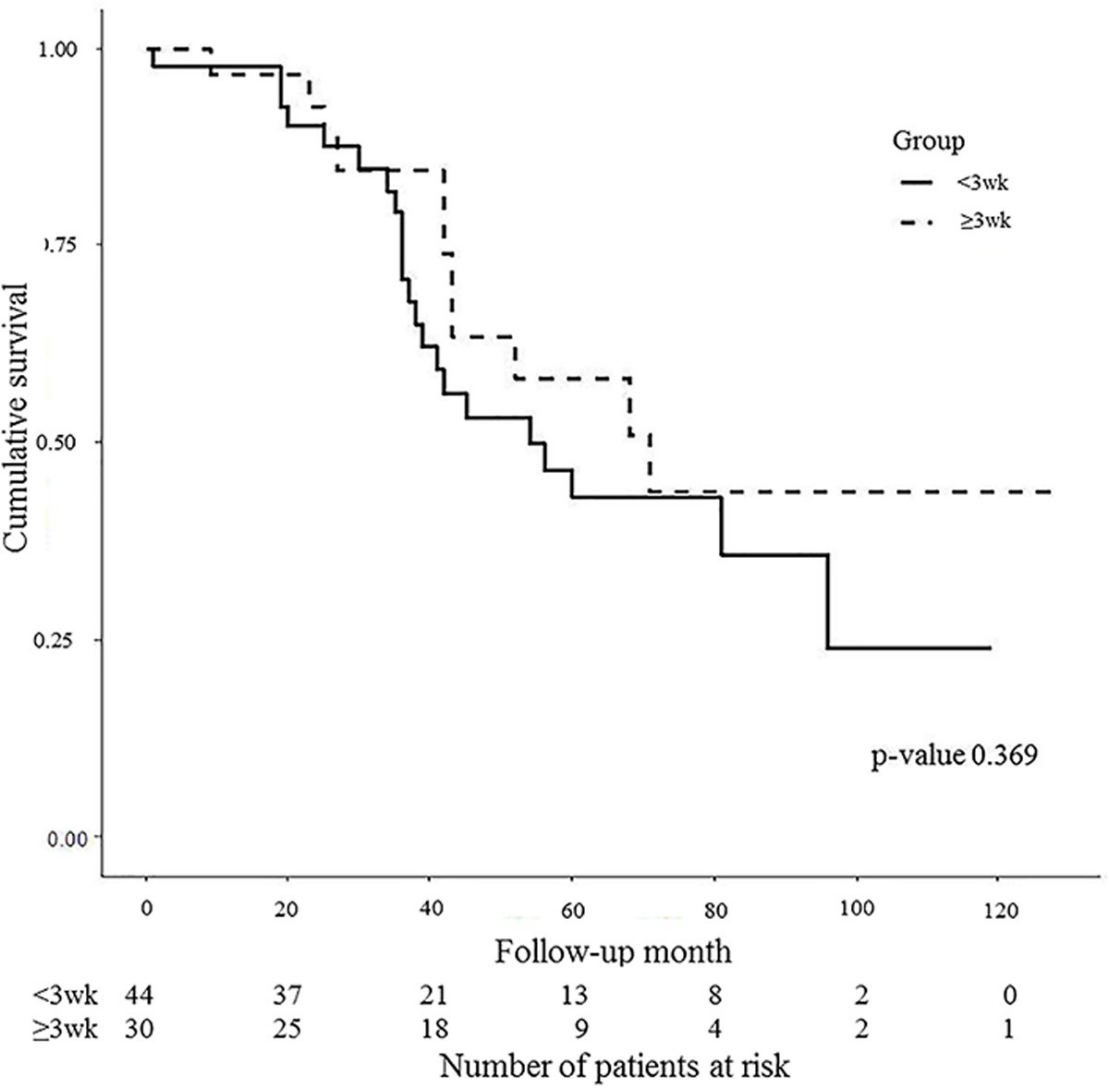
Survival rates of the two groups. Follow-up month (x-axis) indicates the interval from the operation to the last follow-up.

In the ITG, the 3-year and 5-year survival rates were 70.7% and 42.9%, respectively. In the DTG, the 3-year and 5-year survival rates were 84.6% and 58.2%, respectively. No significant benefit in survival rates was observed in the ITG (p = 0.369) (Fig 2).

## Discussion

In this study, there was no statistically significant difference in the total number of chemotherapy regimens versus the total number of chemotherapy cycles following initiation of the third line. However, the total number of regimens and cycles was lower in the DTG than in the ITG. Overall, survival and toxicity did not differ significantly between the ITG and DTG.

For treatment of ovarian cancer recurrence physician decision-making is important. The decision on the chemotherapy regimen or start of treatment might vary depending on the physician’s inclination. At our centers, physicians who opt for aggressive treatment despite multiple relapses generally administer chemotherapy within 2 weeks of recurrence, diagnosed by radiologic or chemical findings. In the palliative setting, the survival benefit of aggressive chemotherapy has been questioned. Rustin et al. found that there was no survival benefit in asymptomatic recurrence with raised CA125 concentration [6]. The National Comprehensive Cancer Network guidelines recommend participation in clinical trials, palliative care, or treatment for recurrence in patients who are platinum-resistant or refractory to treatment [7]. However, the timing of treatment initiation and type of chemotherapy regimen has not been standardized.

A retrospective study compared chemotherapy and supportive care in patients with platinum-resistant ovarian cancer after the first disease progression. The authors concluded that chemotherapy conferred no survival benefit over supportive care [4]. Another study also concluded that aggressive care did not result in improved survival. They found a trend towards increased chemotherapy and overall aggressiveness of care in patients with short survival durations [8]. Our results were consistent with the findings from these studies.

Despite the lack of evidence of a survival benefit with aggressive treatment, patients chose to continue chemotherapy. Some possible explanations for this are the expanding range of chemotherapeutic options, increasing optimism amongst cancer specialists, anecdotal experiences of late‐line treatment success, high expectations and demands from patients and their families, and complexities encountered while communicating a poor prognosis to a patient [9]. One study found that a significant number of patients with gynecologic cancer received chemotherapy or radiotherapy during their last 6 months of life [10]. In Canada, Barbera et al. reported on end of life care for women with gynecologic cancers. Five end of life patterns were observed: receiving chemotherapy, visiting the emergency department, receiving house calls from a physician, receiving a home care visit, and dying in an acute care bed. They reported that patients who died in institutional settings had numerous unmet needs in relation to symptom control, communication, and emotional support. They recommended interventions for improving the end of life care for women with gynecologic cancers [11]. Another study concluded that the family members of patients who received at-home care with hospice services were more likely to report favorable dying experiences [12].

Our study has some limitations. First, we did not evaluate the data pertaining to the targeted agent used for chemotherapy. The current trend in the treatment for ovarian cancer is the use of targeted agents. Furthermore, we could not review non-chemotherapy interventions, such as oral hormonal therapy, that may have contributed to the timing of chemotherapy. Second, the assessment of recurrence was based only on radiologic findings. For evaluation of cancer recurrence, most clinicians use a combination of serial physical examinations, serum markers, and imaging studies [13]. Utilizing these modalities, many patients are diagnosed with recurrence prior to symptom development. The bias towards early diagnosis occurs because it lengthens the follow-up period after diagnosis. This is called lead time bias [14]. In our study, patients in the DTG were only followed-up without treatment for a period of time after recurrence diagnosis. Furthermore, no significant difference in survival was observed between the two groups, which could explain the lead time bias. Third, toxicity could not be fully evaluated due to the retrospective nature of our study. Specifically, if side effects were observed at any point throughout the treatment period, the patient was considered to be exhibiting toxicity. In Japan, Katsumata et al. reported the adverse events associated with each chemotherapy regimen [15]. Compared to their findings, the frequency of hematologic toxicities was lower in our study, while the frequency of sensory neuropathy was higher. Furthermore, there was insufficient information about patient quality of life during treatment. The only data available was the performance status evaluation performed at the initial diagnosis.

A final limitation was the small sample size and retrospective nature of our study. Therefore, further research is needed to corroborate our results. To design a prospective study based on our results, 271 patients in each group, for a total of 542, with 128 months’ follow-up would be needed to detect a difference in survival between the two groups based on 80% power, and at a two-sided significance level of 0.05.

Because recurrent ovarian cancer is a complex condition with several treatment options, patients require sufficient explanation and time to fully understand the costs and benefits related to aggressive chemotherapy, as well as those of supportive hospice-based care. In a palliative care setting, our findings suggest that delaying the treatment of recurrent ovarian cancer has no adverse effect on survival; in other words, it should be acceptable for patients to delay and make informed decisions regarding their treatment. This finding is useful for clinicians with patients who might want to delay starting additional treatment.
